# Metabolic Homeostasis of Immune Cells Modulates Cardiovascular Diseases

**DOI:** 10.34133/research.0679

**Published:** 2025-04-23

**Authors:** Mohan Li, Xiaolei Sun, Linqi Zeng, Aijun Sun, Junbo Ge

**Affiliations:** ^1^ Department of Cardiology, Zhongshan Hospital, Fudan University, Shanghai Institute of Cardiovascular Diseases, Shanghai 200032, China.; ^2^ State Key Laboratory of Cardiology, Zhongshan Hospital, Fudan University, Shanghai 200032, China.; ^3^ Key Laboratory of Viral Heart Diseases, National Health Commission, Shanghai 200032, China.; ^4^ Key Laboratory of Viral Heart Diseases, Chinese Academy of Medical Sciences, Shanghai 200032, China.; ^5^ National Clinical Research Center for Interventional Medicine, Shanghai 200032, China.; ^6^ Institutes of Biomedical Sciences, Fudan University, Shanghai 200032, China.

## Abstract

Recent investigations into the mechanisms underlying inflammation have highlighted the pivotal role of immune cells in regulating cardiac pathophysiology. Notably, these immune cells modulate cardiac processes through alternations in intracellular metabolism, including glycolysis and oxidative phosphorylation, whereas the extracellular metabolic environment is changed during cardiovascular disease, influencing function of immune cells. This dynamic interaction between immune cells and their metabolic environment has given rise to the novel concept of “immune metabolism”. Consequently, both the extracellular and intracellular metabolic environment modulate the equilibrium between anti- and pro-inflammatory responses. This regulatory mechanism subsequently influences the processes of myocardial ischemia, cardiac fibrosis, and cardiac remodeling, ultimately leading to a series of cardiovascular events. This review examines how local microenvironmental and systemic environmental changes induce metabolic reprogramming in immune cells and explores the subsequent effects of aberrant activation or polarization of immune cells in the progression of cardiovascular disease. Finally, we discuss potential therapeutic strategies targeting metabolism to counteract abnormal immune activation.

## Introduction

Cardiovascular disease (CVD) represents a major worldwide health challenge, accounting for the highest mortality rates globally [1]. The escalating prevalence of CVDs, driven by population growth and aging, imposes important health care expenditures and economic impacts at both individual and societal levels.

Accumulating clinical and experimental evidence underscores the pivotal role of systemic and local inflammation in CVD pathogenesis [2]. A persistent inflammatory state exacerbates oxidative stress, thrombosis, and endothelial dysfunction while accelerating fibrosis and maladaptive ventricular remodeling, culminating in the pathogenesis of atherosclerosis, hypertension, and heart failure. Effective immunomodulatory therapies also remain elusive due to a limited understanding of underlying mechanisms.

Therefore, the emerging field of immunometabolism offers a promising avenue for therapeutic intervention by elucidating the role of metabolic reprogramming in regulating immune cell function and differentiation [3]. Originally conceptualized by Mathis and Shoelson in 2011 as the interplay between inflammatory processes and metabolic dysregulation, this field has evolved to delineate how key metabolic pathways regulate immune cell ontogeny, fate, and function [4–6]. The burgeoning interest in immunometabolism presents in a variety of disease areas, including CVD, oncology, neurological disorders, renal disorders, and metabolic disorders [7]. Given the inflammatory milieu, ischemic and hypoxic microenvironment, and lipid metabolic stress in CVD, exploring the therapeutic potential of immunometabolism is imperative.

This review delves into the metabolic reprogramming of immune cells within the context of various CVDs, investigating its impact on immune function and disease progression. Furthermore, we highlight potential targets derived from immunometabolic insights to advance CVD prevention and treatment strategies.

## Overview of Immunometabolism

The past two decades have witnessed the establishment of immunometabolism, advancing our understanding of the bidirectional regulation between cellular metabolic processes and immunological responses [8]. This dynamic interplay encompasses not only the intrinsic metabolic pathways of immune cells but also their interactions of immune cells with the external metabolic and tissue environment [9,10]

Innate immune cells, such as macrophages and dendritic cells (DCs), undergo metabolic reprogramming in response to external microenvironmental stimuli. During microbial challenges, pattern recognition receptors (PRRs) trigger metabolic rewiring in these cells, driving macrophages polarization toward a pro-inflammatory state (also known as classically activated macrophages or M1). This phenotype is characterized by enhanced glycolytic flux and aerobic glycolysis, resembling the “Warburg effect” in neoplastic proliferation. While inefficient in adenosine 5′-triphosphate (ATP) production, this metabolic shift supports rapid cell proliferation [11]. Conversely, in response to stimulation with interleukin-4 (IL-4) or IL-13, macrophages are activated to an anti-inflammatory phenotype (also known as alternatively activated macrophages or M2) and rely on oxidative phosphorylation (OXPHOS) and fatty acid oxidation (FAO). These macrophages maintain robust tricarboxylic acid (TCA) cycle activity while increasing OXPHOS and ATP levels [12].

Macrophage metabolic reprogramming is further exemplified by two critical disruptions of the TCA cycle [13]. First, reduced mRNA transcription of isocitrate dehydrogenase (IDH) leads to citric acid accumulation, diverting this metabolite toward fatty acid synthesis and itaconate production. Itaconate, an anti-inflammatory factor, inhibits succinate dehydrogenase (SDH) and activates Nrf2 [14]. Second, impaired activity of SDH, an enzyme that catalyzes the oxidation of succinate to fumarate, leads to succinate accumulation. This metabolic perturbation stabilizes hypoxia-inducible factor-1α (HIF-1α), increases mitochondrial reactive oxygen species (MtROS), and promotes aerobic glycolysis [15,16].

T cell immunometabolism has garnered significant attention due to its profound therapeutic potential. While resting T cells primarily utilize glucose and fatty acids, activated effector T cells are characterized by up-regulation of glucose transporter (GLUT1), enhanced glucose uptake, and glycolysis to meet their metabolic demands [17]. In contrast, memory T cells predominantly depend on mitochondrial metabolism and FAO [16], whereas regulatory T cells (T_regs_) exhibit a distinct metabolic profile, functioning independently of glucose or amino acid transporters and instead relying mainly on mitochondrial lipid, pyruvate, and lactate oxidation. Fatty acid binding protein 5 (FABP5) expression plays an important role in maintaining mitochondrial integrity and regulating T_reg_ function [18].

B cell metabolism is also tightly regulated and undergoes dynamic reprogramming during development, differentiation, and activation processes [19]. Upon engagement with antigenic and costimulatory signals, B cells increase glucose uptake and aerobic glycolysis for rapid ATP production required in cell proliferation and antibody production [20,21]. Additionally, B cells also up-regulate the expression of endogenous ATP-citrate lyase (ACLY), a key enzyme that drives de novo fatty acid synthesis. This metabolic adaptation increases the production of intracellular lipids, such as cholesterol, free fatty acids, and phospholipids, to support B cell proliferation and plasma cell functional maturation [22]. Memory B cells exhibit a unique metabolic profile that is mainly regulated by the HIF-1α and mammalian target of rapamycin (mTOR) pathways [19], while mTOR signaling pathway plays a pivotal role in regulating germinal center formation in B cells and is essential for facilitating antibody class switching reactions [21,23]. Blocking mTOR in the activated B cell response leads to a bias in antibody production toward immunoglobulin M (IgM) [24], highlighting the impact of metabolic reprogramming on antibody response.

As central hubs of cellular energy metabolism, mitochondria not only directly shape immune cell phenotypes and function through their metabolic and biosynthetic activities but also serve via an indirect regulatory mechanism through mitochondrial dynamics. Mitochondrial dynamics refers to a dynamic regulatory network governing morphology, quality, and function to maintain metabolic adaptability [25,26]. Studies reveal that dynamin-related protein-1(Drp1) deficiency in macrophages suppresses mitochondrial fission, enhancing glycolysis to promote M1 polarization [27]. In T cells, memory T cells highly depend on OXPHOS, and thus, they require cristae integrity. Loss of fusion protein optic atrophy 1 (OPA1) disrupts their function, while effector T cells up-regulate DRP1 to fragment mitochondria and facilitate glycolysis [27]. These findings collectively demonstrate the integral role of mitochondrial metabolism in immune cell homeostasis, highlighting its therapeutic potential as a target for immunometabolism.

In summary, immune cell metabolism is highly diverse and tailored to specific cellular functions and microenvironmental cues. Understanding these differences provides a foundation to elucidating the pathogenesis of inflammation-related diseases and developing targeted therapeutic interventions to modulate immune homeostasis.

## Immunometabolic Mechanisms of CVDs

### Atherosclerosis

Atherosclerosis, a chronic inflammatory disorder of the vasculature, represents a leading contributor of CVD [28–30]. Atherosclerotic plaques are characterized by lipid accumulation within the arterial wall, accompanied by sustained recruitment of immune cells, including macrophages, T cells, and mast cells, as well as the deposition of a collagen-based fibrous cap mediated by vascular smooth muscle cells [31].

Over the past two decades, macrophages have been recognized as the protagonists in atherosclerotic plaque development and their thrombotic complications [32]. The dyslipidemic microenvironment within the arterial wall modulates metabolic pathways of macrophages, influencing their immune function through various mechanisms [31,33]. Within the atherosclerotic arterial wall, oxidized low-density lipoprotein (oxLDL) are predominantly internalized by macrophages via scavenger receptors, activating NLRP3 inflammasome and pro-inflammatory cytokines [34]. This process critically exacerbates local inflammation and worsens clinical outcomes in atherosclerosis.

Beyond activating inflammatory signaling, CD36, as a scavenger receptor expressed on the surface of a variety of innate and adaptive immune cells, plays the most crucial role [31,35,36]. CD36 triggers metabolic reprogramming upon oxLDL binding, up-regulating glucose transporter 1 (GLUT1), pyruvate dehydrogenase kinase 1 (PDHK1), and glycolysis rate-limiting genes while down-regulating TCA cycle genes. This metabolic shift is accompanied by an increase in the production of multiple glycolytic enzymes and ROS, which facilitates macrophage differentiation toward a pro-inflammatory phenotype [31,37]. Fatty acid transport function of CD36 also contributes to mitochondrial metabolism reprogramming by facilitating long-chain fatty acid (LCFA) import, accumulation, and FAO inhibition [31]. Additionally, oxLDL inactivates acetyl coenzyme A carboxylase 2 (ACC2) in a CD36-independent manner, thereby increasing LCFA transport to mitochondria by decreasing the inhibition of carnitine palmitoyltransferase-1 (CPT1) [31]. Mitochondrial accumulation of LCFA also induces ROS production, potentially through LCFA-mediated inhibition of the electron transfer chain (ETC) complex, which subsequently promotes reverse electron transfer [38]. MtROS production can lead to oxidative stress that inactivates multiple enzymes in the TCA cycle and disrupts the ETC. MtROS can also promote macrophages to secrete pro-inflammatory cytokines and reprogram metabolism [39].

Hypoxia, another key factor in the plaque microenvironment, significantly contributes to atherogenesis [40–42]. Under hypoxia, macrophages and foam cells increase glucose uptake, diverting glucose toward lactate production rather than TCA cycle metabolism. HIF-1α, a major regulator of hypoxic responses, induces up-regulation of GLUT1 and glycolytic enzymes, increasing glycolytic flux and limiting OXPHOS to promote the shift of macrophages toward a pro-inflammatory phenotype. Consequent citrate and succinate accumulation due to TCA cycle disruption contributes to adipogenesis, pro-inflammatory mediator production, and MtROS generation [11,16]. Meanwhile, succinate also stabilized HIF-1α, increasing HIF-1α-dependent IL-1β secretion [16].

Furthermore, emerging experimental evidence derived from both human atherosclerotic monocytes and mouse models indicates that systemic pathological stimuli, such as lipoproteins, glucose, and diet, have modulated cellular metabolism and function before the arrival of monocytes at the arterial wall. It allows the monocytes to begin their transformation to an inflammatory phenotype before they differentiate into macrophages [32,41]. Monocytes isolated from atherosclerotic patients exhibit increased extracellular acidification rate (ECAR), glycolytic fluxes, and pro-inflammatory cytokine production compared with healthy controls [43]. This pre-activated phenotype persists upon differentiation into macrophages, exacerbating atherosclerosis development.

In addition to metabolic reprogramming being an integral part of regulating the activation response of macrophages and monocytes, recent studies have also revealed its role in innate immune training [32,41]. Innate immune memory (also known as trained immunity) represents an adaptive mechanism where prior exposure to certain pathogens or vaccines can induce enhanced nonspecific protection of innate immune cells, including monocytes, macrophages, and natural killer cells, against secondary infections [41,44]. Within the pathophysiology of CVD, trained immunity can also be primed by endogenous proatherogenic stimuli, notably oxLDL [29,32]. It has been demonstrated that isolated human monocytes can induce a pro-inflammatory macrophage phenotype after transient stimulation for oxLDL and can increase the production of tumor necrosis factor-α (TNF-α) and IL-6 upon restimulation. Mechanistically, this enhancement can be abolished by the pharmacological blockade of histone methylation through administration of the nonspecific methyltransferase inhibitor 5′-methylthioadenosine [29,45]. This suggests an important epigenetic role in oxLDL-induced training immunity. Interestingly, TCA cycle intermediates, acting as cofactors and substrates of epigenetic enzyme, contribute to this process. For instance, β-glucan-stimulated macrophages exhibit increased glucose uptake rate, glycolysis, and accumulation of fumarate. The accumulation of fumarate induces histone modifications, including increased H3 histone lysine 4 trimethylation (H3K4me3) expression and inhibition of the KDM5 family of histone demethylases, which enhances macrophage innate immune training [44,46]. Similarly, oxLDL and hypoxia in the atherosclerotic microenvironment induce metabolic reprogramming in macrophages and monocytes, increasing glycolytic flux while limiting OXPHOS, disrupting the TCA cycle, and leading to persistent epigenetic modifications, which promotes immune gene expression.

**Fig. 1.**
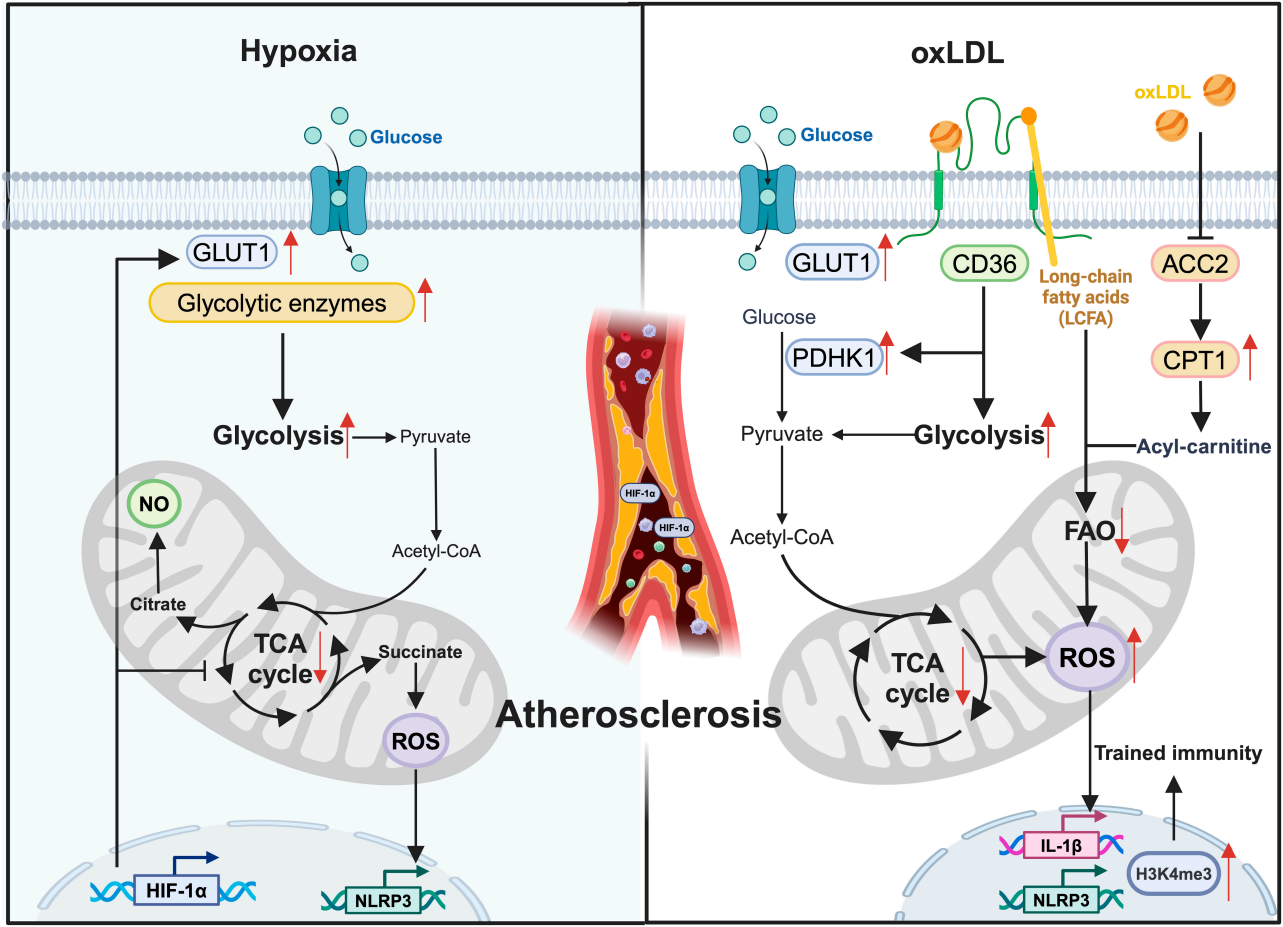
Immunometabolic mechanisms in atherosclerosis. HIF-1α induces up-regulation of GLUT1 and glycolytic enzymes and consequent citrate and succinate accumulation under hypoxia. In addition, CD36 up-regulates GLUT1 and PDHK1 upon oxLDL binding and facilitates the accumulation of long-chain fatty acid (LCFA).

In conclusion, the microenvironment of atherosclerotic plaques, characterized by oxLDL accumulation and hypoxia, orchestrates profound metabolic alterations in immune cells, particularly macrophages and monocytes (Fig. 1). The metabolic reprogramming from OXPHOS to glycolysis in macrophages and monocytes impacts cellular function, phenotypic plasticity, and epigenetic memory, ultimately exacerbating atherogenesis and inflammation.

### Hypertension

Hypertension, clinically defined as persistently elevated systolic blood pressure (SBP) exceeding 130 mmHg and/or diastolic blood pressure (DBP) exceeding 80 mmHg, represents a significant worldwide public health burden. Accumulating evidence has established a critical link between dysregulated inflammatory responses and the pathogenesis of hypertension and associated complications [47].

The sympathetic nervous system is one of the regulators of macrophage activity in tissues. Sympathetic nerve endings in tissues secrete norepinephrine (NE), which then transmits signals to macrophages via β2-adrenergic receptors (B2AR) [48]. NE-induced metabolic reprogramming in macrophages involves increased triglyceride synthesis and reduced triglyceride esterolysis via adipose triglyceride lipase (ATGL) inhibition, leading to triglyceride accumulation. This lipid accumulation suppresses FAO, promoting a pro-inflammatory phenotype and cytokine secretion, including IL-1β and IL-6 [49]. Infiltration of inflammatory cytokines into the vasculature and kidneys exacerbates vascular inflammation, microvascular remodeling, and renal dysfunction, contributing to the progression of hypertension [47,48].

In hypertension conditions, renin–angiotensin–aldosterone system (RAAS) activation serves as an important modulator of oxidative stress and the immune system. Angiotensin II (Ang II), a key effector peptide of the RAAS, stimulates ROS generation through up-regulation of nicotinamide adenine dinucleotide phosphate oxidases (NOXs) in both endothelial and immune cells. This oxidative stress leads to pro-inflammatory mediator production, immune cell metabolic reprogramming, and systemic immune activation [47]. Activated NOX-induced vascular ROS enhance T cell receptor (TCR) signaling, activating nuclear factor of activated T cells (NFAT) and c-MYC, promoting a metabolic shift toward aerobic glycolysis [50–52]. Activated T cells infiltrate tissues and produce cytokines, such as IL-17, which contribute to renal and vascular dysfunction and lead to end-organ damage in hypertension [53].

In addition, aldosterone and catecholamines induce trained immunity in immune cells [54,55]. Aldosterone binds to the salicorticoid receptor in macrophages, up-regulating fatty acid synthesis genes and pro-inflammatory cytokines, including IL-6, and TNF-α [54]. Epinephrine and NE induce both glycolysis and OXPHOS in monocytes via the β-adrenergic receptors, with further enhancement in differentiated macrophages accompanied by increased secretion of TNF-α and IL-8 [55]. In addition, aldosterone and catecholamines have also been found to induce enrichment of H3K4me3, which alters the epigenetic network of metabolic reprogramming in monocytes/macrophages and enhances the transcription of inflammatory genes [54,55].

Excessive dietary salt intake elevates cardiac output, impairs vascular compliance, and increases systemic vascular resistance [56]. At the cellular level, studies have shown that$\;{\mathrm{Na}}^{+}\;$can enter the mitochondrial matrix through the$\;{\mathrm{Na}}^{+}/{\mathrm{Ca}}^{2+}$ exchanger (NCLX) and interact with phospholipids in the inner mitochondrial membrane, which reduces mitochondrial membrane fluidity and ubiquinone-mediated electron transfer between electron transport chain complexes II and III, ultimately impeding OXPHOS efficiency [56,57]. Additionally, key enzymes of the TCA cycle, such as SDH, IDH, and malate dehydrogenase, are down-regulated in T cells exposed to excessive salt, with accumulation of succinate further inhibiting OXPHOS [57]. This metabolic shift drives functional activation of macrophages, monocytes, and T cells while impairing the function of T_regs_, which promotes pro-inflammatory cytokine secretion and accelerates hypertension progression.

In summary, the complex interplay of hypertension-related risk factors drives metabolic reprogramming within immune cells, resulting in polarization toward pro-inflammatory phenotypes and pro-inflammatory cytokine release. Excessive and chronic inflammatory responses ultimately propagate irreversible vascular endothelial cell damage, organ dysfunction, and fibrosis, contributing to complications of hypertension [47].

### Heart failure

Heart failure represents a progressive, life-threatening clinical syndrome characterized by high morbidity and mortality, significant healthcare burden, and a profound decline in patient quality of life [58,59]. To facilitate exploration of immunometabolic mechanisms and potential therapeutic targets, heart failure is commonly categorized into two primary subtypes based on differing etiologies and pathophysiological processes: reduced ejection fraction (HFrEF) and preserved ejection fraction (HFpEF).

### HFrEF

HFrEF is a common complication of myocardial infarction (MI) [60,61]. Metabolic shift of immune cells in post-MI microenvironment has a profound impact on the progression of HFrEF.

Following MI, the release of damage-associated molecular patterns (DAMPs) and autoantigens from necrotic cardiomyocytes triggers metabolic reprogramming in neutrophils, macrophages, T cells, and B cells through different Toll-like receptors (TLRs) [62,63]. Neutrophils and monocytes, as early responders recruited, undergo HIF-1α-mediated metabolic reprogramming in response to the hypoxic microenvironment and TLR stimulation, shifting toward glycolysis and producing inflammatory cytokines [62,64]. Macrophages after experimental MI also exhibit pro-inflammatory phenotypes, demonstrating elevated transcription of glycolytic genes consistent with the biosynthesis of inflammatory cytokines and hypoxic adaptation [62,65].

After experimental MI, CD8^+^ T cell depletion ameliorated pathological remodeling and cardiac dysfunction, highlighting the role of these cells in disease progression. CD8^+^ T cell function required support from elevated GlUT1 expression and increased glycolytic metabolism. In addition, polyclonal B cells were also recruited to the ischemic myocardium after experimental MI and contributed to cardiac dysfunction by secreting chemokines, such as CCL7, which recruited monocytes, activated inflammatory macrophages, and further impaired cardiac function. Notably, CCL7 is one of the transcriptional targets of HIF-1α, underscoring the importance of glycolysis in T and B cell-mediated inflammation post-MI [62].

Activation of inflammation after acute MI is aimed at removing necrotic cardiomyocytes. A transition from a pro-inflammatory phase to an anti-inflammatory phase is indispensable for myocardial repair and scar formation. During the phase of inflammation abatement, macrophages up-regulate genes associated with mitochondrial metabolism and OXPHOS. Concurrently, itaconate production increases. Itaconate is a bypass metabolite of the TCA cycle and is produced by aconitrate in response to decarboxylase encoded by immune response gene 1 (IRG1) [65]. Itaconate antagonizes SDH to attenuate inflammation and ROS production [62,65]. Persistent dysregulated inflammation, resulting from inadequate anti-inflammatory responses, exacerbates cardiomyocyte death, impairs contractile function, and increases cardiac workload, ultimately leading to heart failure [61,66].

### HFpEF

HFpEF is usually associated with left ventricular diastolic dysfunction [67]. Patients with HFpEF exhibit characteristics of obesity, advanced age, female sex, and comorbidities including hypertension or atrial fibrillation [68].

Obesity is characterized by excessive adipose tissue accumulation and chronic inflammation [69]. Macrophages constitute approximately 60% of adipose tissue cells, predominantly exhibiting an M1 phenotype [70]. Myocardial hypoxia can also be induced by adipose tissues due to capillary laxity and fat accumulation, which activates HIF-1α in adipose tissue macrophages, promoting glycolysis and pro-inflammatory gene expression [71]. Fatty acid-derived small lipid mediators further exacerbate inflammation by inducing M1 macrophage polarization via nuclear factor κB (NF-κB) signaling and cytokine production [72,73]. In addition, adipokines released from adipose tissue influence macrophage polarization. Studies demonstrate that leptin up-regulates glucose uptake and glycolytic enzyme expression in macrophages. It also accelerates cholesteryl ester accumulation in cells and inhibits cholesterol efflux to induce inflammatory phenotype [74,75].

Hypertension represents another critical risk factor for HFpEF, contributing to cardiac fibrosis and insufficiency. Increased cardiac pressure loading induces myocardial hypoxia, shear stress, and vascular endothelium dysfunction [73]. Endothelial dysfunction up-regulates Ang II, leading to NADPH (reduced form of nicotinamide adenine dinucleotide phosphate) oxidase activation and ROS generation [76–78]. Excessive ROS promotes glucose uptake rate and glycolytic flux in macrophages and therefore induces M1 phenotypic polarization and pro-inflammatory cytokine production.

In summary, different etiologic factors and triggers disrupt the delicate balance between the pro-inflammatory and anti-inflammatory immune responses. This immune imbalance leads to cardiac function and ultimately culminates in heart failure (Table 1).

**Table 1.** Comparative analysis of immunometabolic mechanisms in HFrEF versus HFpEF

	HFrEF	HFpEF
Clinical phenotype	Systolic dysfunction	Diastolic dysfunction
Primary etiology	Myocardial infarction	• Obesity • Hypertension • Advanced age • Female sex
Pathological processes	• Necrotic cardiomyocytes release DAMPs and autoantigens • Persistent dysregulated inflammation	• Chronic adipose tissue inflammation • Myocardial hypoxia • Endothelial dysfunction • Excessive ROS production
Immune cell metabolic reprogramming	• Neutrophils/monocytes: HIF-1α-mediated glycolysis up-regulation and pro-inflammatory cytokine secretion • Macrophages: Pro-inflammatory polarization and elevated transcription of glycolytic genes • CD$8^{+}$ T cells: Elevated GlUT1 expression and increased glycolytic metabolism • Polyclonal B cells: Secreting chemokines, such as CCL7	• Adipose tissue macrophages: HIF-1α-driven glycolysis up-regulation and pro-inflammatory gene expression • Circulating macrophages: ROS-induced glycolysis, elevated glycolytic enzyme expression, and M1 phenotypic polarization
Immunometabolic mechanisms	• TLR/DAMP signaling • HIF-1α-mediated glycolysis • IRG1–itaconate–SDH-induced anti-inflammatory pathway	• Adipokine leptin-driven metabolic reprogramming • Ang II–NADPH oxidase–ROS pathway

### Diabetic heart disease

Diabetes mellitus, a progressive metabolic disorder characterized by chronic hyperglycemia, arises from insulin deficiency and/or resistance [79]. Diabetes patients exhibit increased incidence of heart failure [80,81]. It is suggested that altered levels of metabolic substrates in the context of hyperglycemia affect macrophage polarization and function. Monocytes and macrophages respond to elevated external glucose levels and up-regulate the surface density of GLUT1 to dynamically adapt their glycolytic machinery. Glucose-rich microenvironments can contribute to the inflammatory phenotype of monocytes and macrophages, leading to chemokine and cytokine production [73,82].

Hyperglycemia activates the nicotinamide adenine dinucleotide (NADH) oxidase system and increases mitochondrial oxygen radical production [83]. Glucose-induced ROS directly induce a pro-inflammatory phenotype in macrophages. Additionally, ROS contribute to advanced glycation end-product (AGE) formation via methylglyoxal (MGO) generation [84–86]. It has been shown that binding of AGEs to their receptor RAGE increases the expression of the interferon gene IRF7. Knockdown of IRF7 in bone marrow-derived macrophages (BMDMs) not only attenuates the pro-inflammatory effects of macrophages but also leads to the up-regulation of cholesterol transporters and the down-regulation of CD36, which facilitates cholesterol efflux [87,88]. Intracellular cholesterol accumulation damages the cell membrane, leading to endoplasmic reticulum and mitochondrial stress, further exacerbating macrophage inflammation and atherogenesis [41]. The highly pro-inflammatory environment leads to a range of cardiac pathologies including myocardial ischemia, reduced ejection fraction, and finally heart failure [89,90].

**Fig. 2.**
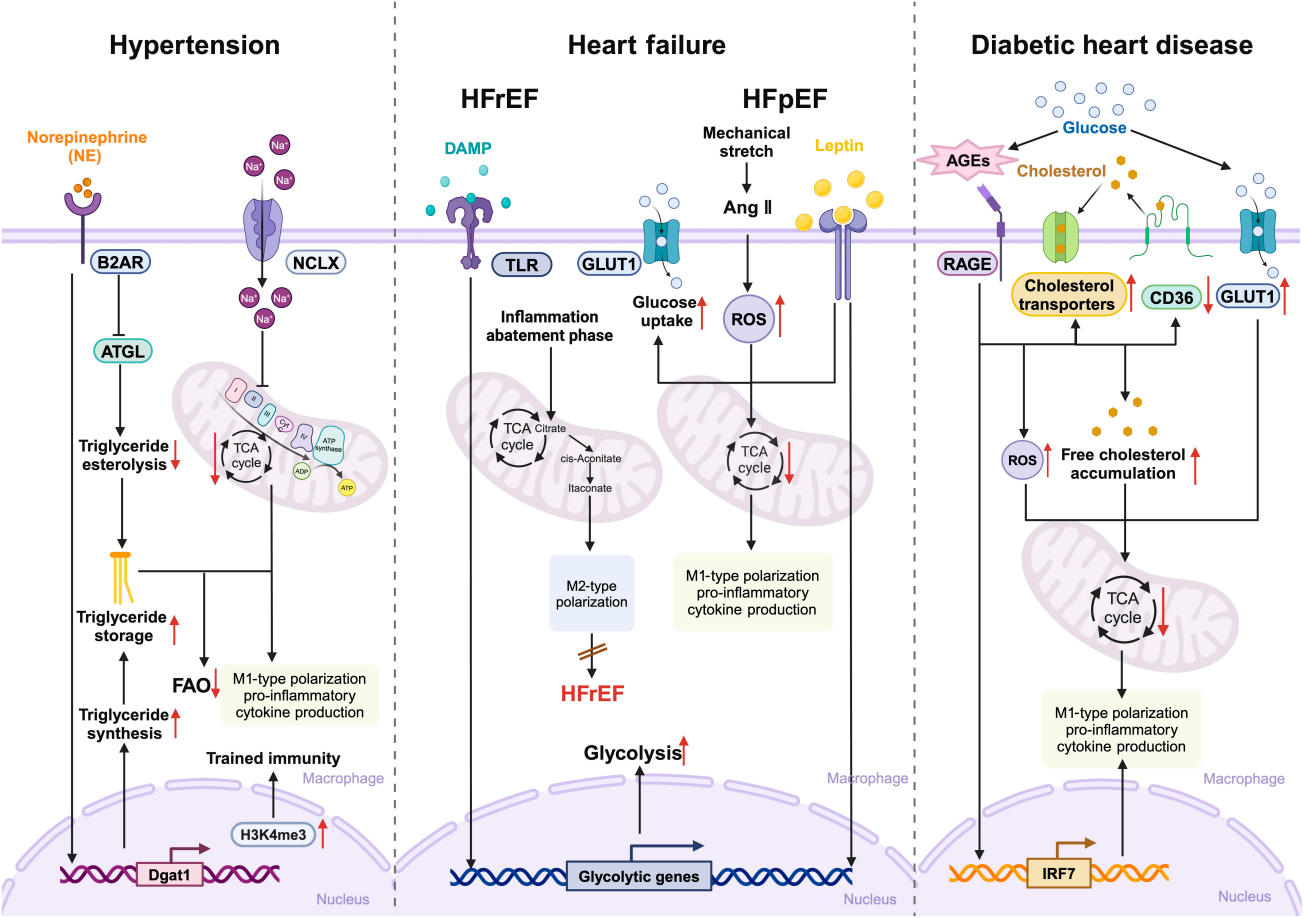
Immunometabolic mechanisms in other cardiovascular disease. The complex interplay of risk factors and continuous changes in the microenvironment stimulate metabolic changes in macrophages, leading to the secretion of pro-inflammatory cytokines.

Overall, changes in systemic circulating substrates during diabetes induce metabolic reprogramming and inflammatory phenotypes in immune cells. Targeting these immunometabolic pathways may offer novel therapeutic strategies for diabetic heart disease (Fig. 2).

## Therapeutic Strategies Targeting Immune Metabolism

Given the intricate interplay between metabolic and inflammatory processes, therapeutic strategies that target metabolism hold significant promise. However, the development of therapeutic approaches specifically designed to modulate immune metabolism remains in its infancy.

### Dimethyl fumarate

Dimethyl fumarate (DMF), as a product derived from Krebs cycle intermediates, have been shown to affect immune cell metabolism [91–93]. Clinically approved for treating psoriasis and multiple sclerosis, DMF exerts its anti-inflammatory effects primarily through cysteine residue succinylation [94]. With glycolytic enzyme glyceraldehyde-3-phosphate dehydrogenase (GAPDH) as its main target, DMF can inactivate GAPDH, inhibit aerobic glycolysis in immune cells, and prevent immune activation toward anti-inflammatory phenotype [95]. Studies have shown that DMF can regulate glucose metabolism in myocardial macrophages, promoting an anti-inflammatory phenotype of macrophages. Therefore, DMF can improve ventricular wall remodeling, collagen deposition, and angiogenesis after left ventricular MI [96]. Studies in MI models also reveal its ability to attenuate collagen deposition in infarcted zones and reduce infarct size [96]. In diabetic animal models, DMF treatment mitigates adverse myocardial injury outcomes [97]. Overall, these findings underscore that DMF has a cardioprotective potential in CVDs [98].

### Itaconate

Itaconate, an immunometabolite with anti-inflammatory properties, is generated through the decarboxylation of cis-aconitate within the TCA cycle. This biochemical conversion is catalyzed by cis-aconitate decarboxylase (CAD) encoded by IRG [99,100]. Due to its structural similarity to succinate, itaconate can act as a competitive inhibitor of SDH and play an immunomodulatory role in macrophages [100]. SDH serves as a critical regulator governing macrophage polarization, which drives MtROS generation and induces pro-inflammatory gene expression through enhanced succinate oxidation and mitochondrial membrane hyperpolarization [16]. Conversely, itaconate-mediated inhibition of SDH exerts an anti-inflammatory effect, characterized by down-regulation of pro-inflammatory factor IL-1β and IL-6 expression, alongside up-regulation of anti-inflammatory factors IL-1RA and IL-10. Similar to DMF, itaconate can also modify the cysteine sites of macrophage proteins, including GAPDH, fructose diphosphate acetylase A (ALDOA), and lactate dehydrogenase A (LDHA). It significantly inhibits the catalytic activity of glycolytic enzymes and glycolytic pathway in macrophages, thus exerting anti-inflammatory effects [101,102]. Studies demonstrate that itaconate can reduce infarct size in murine MI models and confirmed its therapeutic efficacy in doxorubicin-induced cardiotoxicity, highlighting its therapeutic prospects for CVDs [103,104].

### Metformin

Metformin is widely used in the treatment of diabetes due to its ability to enhance insulin sensitivity. Extensive research has demonstrated that metformin not only modulates glucose-lipid homeostasis but also suppresses the expression of various pro-inflammatory factors [105,106]. These anti-inflammatory properties may result in the inhibition of mitochondrial respiratory chain complex I, which induces cellular energy stress and intracellular alterations of energetic substances. Subsequently, the altered adenosine 5′-monophosphate (AMP)/ATP ratio triggers the activation of AMP-activated protein kinase (AMPK) [107]. AMPK subsequently activates Nrf2 and inhibits signaling pathways such as c-Jun N-terminal kinase (JNK) and signal transducer and activator of transcription 3 (STAT3) [108–110]. It has been reported that metformin exerts an inhibitory effect on the differentiation from monocytes into macrophage within the THP1 macrophage cell line. This mechanism appears to be mediated through the activation of AMPK, which subsequently suppresses STAT3 signaling. It is beneficial in attenuating atherosclerosis by reducing inflammation in the vessel wall [110]. AMPK also down-regulates the expression of adipose synthesis genes by modulating transcriptional regulators, including sterol regulatory element binding protein-1c (SREBP-1c) and carbohydrate response element binding protein (ChREBP). They inhibit de novo lipogenesis while enhancing fatty acid β-oxidation, thereby facilitating macrophage polarization to an anti-inflammatory M2 phenotype [111–113]. Therefore, metformin offers a potential anti-inflammatory therapeutic strategy to mitigate cardiovascular injury in diabetes by activating the AMPK/Nrf2 pathway [114].

In addition to agents mentioned above, rapamycin is also recognized to inhibit the mTORC1 pathway to suppress glycolysis and attenuate macrophage inflammatory polarization, thereby mitigating post-MI inflammatory macrophage infiltration and ameliorating adverse outcomes [115].

In summary, a variety of drugs targeting metabolism demonstrate significant potential in regulating immune responses (Table 2). Further elucidating the underlying mechanisms in immune responses will significantly unveil potential therapeutic targets, thereby facilitating the development of innovative intervention strategies for both CVD management and prophylaxis.

**Table 2.** Drugs targeting immune metabolism

Drugs	Target(s) in immune cells	Inflammatory outcome(s)
Dimethyl fumarate (DMF)	• Succinylate cysteine residues • Inactivate glyceraldehyde-3-phosphate dehydrogenase (GADPH)	• Down-regulate aerobic glycolysis in immune cells • Prevent immune activation • Promote the anti-inflammatory/repair phenotype of macrophages
Itaconate	• Act as a competitive inhibitor of SDH • Modify the cysteine sites of macrophage proteins • Inhibit the catalytic activity of glycolytic enzymes	• Block the production of pro-inflammatory factors IL-1β and IL-6 • Enhance the levels of anti-inflammatory factors IL-1RA and IL-10
Metformin	• Inhibit mitochondrial respiratory chain complex I and trigger the activation of AMPK • Reduce the expression of adipose synthesis genes • Prevent monocyte-to-macrophage conversion in the THP1 macrophage cell line	• Activate Nrf2 and inhibit JNK and STAT3 signaling pathways • Inhibit fatty acid synthesis and stimulate enzymes involved in fatty acid β-oxidation • Facilitate the transition of macrophages to the M2 phenotype

## Conclusions and Perspectives

The concept of immunometabolism reveals how alterations in metabolic substrates, oxygen availability, and neuroendocrine factors in the myocardial microenvironment regulate the metabolic reprogramming of immune cells, ultimately affecting cellular immune function and polarization. Radiolabeled tracer-based positron emission tomography (PET) scans offers a promising approach to visualize altered metabolic states at the cellular and subcellular levels [116].

Additionally, imaging techniques employing defined metabolic markers can be utilized to dissect the relationship between immunity and metabolism [117]. The combination of metabolomics, proteomics, and transcriptomics further enables the analysis of dynamic metabolite fluctuations [118–120]. However, it is noteworthy that current studies in macrophages predominantly adhered to the M1/M2 polarization paradigm, which oversimplified the complexity of immune cell heterogeneity in vivo. Further subdivision is needed to investigate the role of immune metabolism across diverse cardiac macrophage subpopulations and in various disease contexts.

Preclinical and clinical studies have validated the cardioprotective effects of modulating immunometabolic pathways. However, extensive targeting may trigger off-target effects due to the complex roles of immune cells. Thus, future research should focus on characterizing disease-specific immune cell subsets and their metabolic phenotypic heterogeneity in CVDs. With the continuous advancement of gene-editing technologies, tools such as CRISPR-Cas9 and base editing provide novel approaches to precisely dissect the immunometabolic regulatory network. Targeted editing of key metabolic genes in immune cells serves as a critical approach to intervening in immunometabolic processes and modulating immune cell differentiation and functional polarization. Developing targeted delivery systems is essential to spatially restrict metabolic modulators to lesion sites. Preclinical and clinical data should also be integrated to refine therapeutic efficacy and safety profiles.

In conclusion, cardiac immunometabolism remains a nascent field. Given the critical role of systemic and cardiac metabolism–inflammatory crosstalk in driving the development of CVD, this area warrants substantial investigation with interdisciplinary collaboration and comprehensive techniques.
